# The Aurora B gradient sustains kinetochore stability in anaphase

**DOI:** 10.1016/j.celrep.2021.109818

**Published:** 2021-11-09

**Authors:** Diana Papini, Mark D. Levasseur, Jonathan M.G. Higgins

**Affiliations:** 1 Biosciences Institute, Faculty of Medical Sciences, Newcastle University, Newcastle upon Tyne NE2 4HH, UK

**Keywords:** mitosis, chromosome segregation, centromere, kinetochore, anaphase, protein kinase, Aurora B, Cdk1, Astrin, Dsn1

## Abstract

Kinetochores assemble on chromosomes in mitosis to allow microtubules to attach and bring about accurate chromosome segregation. The kinases Cyclin B-Cdk1 and Aurora B are crucial for the formation of stable kinetochores. However, the activity of these two kinases appears to decline dramatically at centromeres during anaphase onset, precisely when microtubule attachments are required to move chromosomes toward opposite poles of the dividing cell. We find that, although Aurora B leaves centromeres at anaphase, a gradient of Aurora B activity centered on the central spindle is still able to phosphorylate kinetochore substrates such as Dsn1 to modulate kinetochore stability in anaphase and to regulate kinetochore disassembly as cells enter telophase. We provide a model to explain how Aurora B co-operates with Cyclin B-Cdk1 to maintain kinetochore function in anaphase.

## Highlights

• Central spindle Aurora B targets kinetochore substrates in anaphase
• Phosphorylation of Dsn1 by Aurora B stabilizes kinetochores in anaphase
• Dsn1 phosphorylation modulates chromosome movements in anaphase

## Graphical abstract

**Figure:**
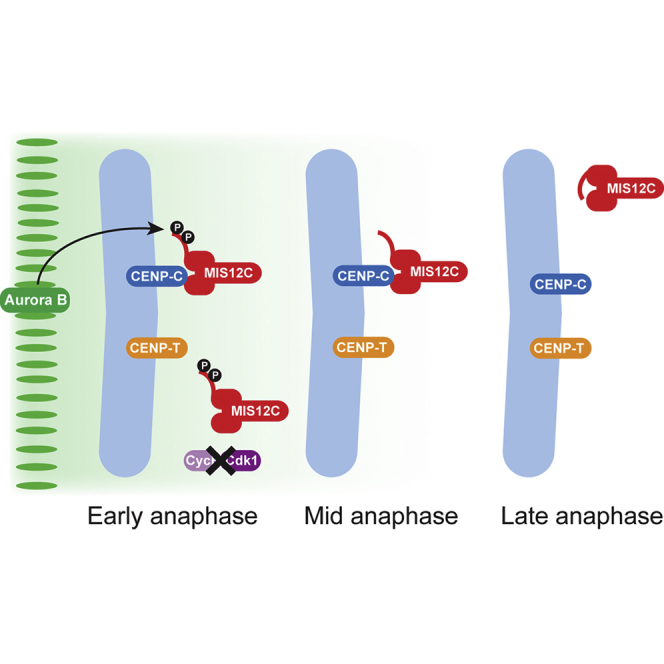

## Teaser

Kinetochores assemble on chromosomes in mitosis to allow accurate chromosome segregation. Papini et al. find that, although Aurora B leaves centromeres at anaphase, an Aurora B activity gradient centered on the central spindle phosphorylates kinetochore substrates such as Dsn1 to modulate kinetochore stability and chromosome movements in anaphase.

## Introduction

The mitotic spindle acts to segregate chromosomes accurately into daughters during cell division. To accomplish this, kinetochores are built on centromeres in early mitosis to enable chromosomes to capture microtubules. Accordingly, the mechanisms that ensure that kinetochores assemble and attach to microtubules emanating from a single pole have been extensively studied. For example, in early mitosis, the kinase Aurora B plays a vital role in both kinetochore assembly and destabilizing incorrect kinetochore-microtubule attachments (Akiyoshi et al., 2013; Emanuele et al., 2008; Kim and Yu, 2015; Rago et al., 2015; Tanaka et al., 2002; Welburn et al., 2010; Yang et al., 2008), and Cyclin B-Cdk1 is also required for key proteins to localize to the kinetochore (Gascoigne and Cheeseman, 2013; Gascoigne et al., 2011; Hara et al., 2018; Huis In ’t Veld et al., 2016; Nishino et al., 2013). In many respects, these activities serve as a prelude to the most obvious function of kinetochores: driving chromosome movement to opposing spindle poles in anaphase, when kinetochore-spindle attachments are known to be stable (Asbury, 2017; Cimini et al., 2004; Zhai et al., 1995). However, it is striking that key processes that control kinetochore assembly and function are driven by the activity of Cyclin B-Cdk1, which strongly declines at the metaphase-to-anaphase transition (Clute and Pines, 1999; Murray et al., 1989), and Aurora B, which dissociates from centromeres and transfers to the central spindle in anaphase (Carmena et al., 2012). For example, the phosphorylation of the KMN (Knl1 complex, Mis12 complex, Ndc80 complex) protein Dsn1 at S100 and/or S109 by Aurora B is required for the Mis12 complex (Mis12C) to stably associate with the constitutive centromere-associated network (CCAN) proteins CENP-C and CENP-T (Akiyoshi et al., 2013; Dimitrova et al., 2016; Hara et al., 2018; Kim and Yu, 2015; Petrovic et al., 2016; Rago et al., 2015; Walstein et al., 2021; Yang et al., 2008). However, it is widely believed that the function of Aurora B at kinetochores ceases in anaphase (Asbury, 2017; Mirchenko and Uhlmann, 2010; Parry et al., 2003; Vázquez-Novelle and Petronczki, 2010). This raises the question of how kinetochore stability is maintained in anaphase, precisely when spindle attachments are critical to move chromosomes. A related and also understudied question is how kinetochores are disassembled as cells enter telophase.

Here we find that the previously reported gradient of Aurora B activity centered on the spindle midzone in anaphase (Fuller et al., 2008) is able to phosphorylate kinetochore substrates to regulate kinetochores in late mitosis.

## Results

### The KMN protein Dsn1 is phosphorylated at kinetochores in early anaphase

In anaphase, Aurora B focused at the central spindle generates a gradient of activity that results in high phosphorylation of substrates at the midzone and progressively lower phosphorylation toward the poles (Fuller et al., 2008). As previously reported, this gradient can be observed in HeLa cells when visualizing phosphorylation of histone H3 at S10 (H3S10ph), a product of Aurora B activity on anaphase chromosomes, particularly when cells were treated with MPS1-IN-1, an inhibitor of the mitotic checkpoint kinase Mps1 (Kwiatkowski et al., 2010), to induce lagging chromosomes (Figure S1A). A gradient of phosphorylation of the centromeric Aurora B target CENP-A S7 was also apparent in anaphase in RPE1 cells (Figure S1B), consistent with the previously observed gradient of phosphorylation on an artificial substrate targeted to centromeres with CENP-B (Fuller et al., 2008). However, despite previous speculation (Emanuele et al., 2008), whether there is a gradient of Aurora B activity that influences kinetochores themselves, as well as whether this has functional consequences, has not been explored.

**Figure 1:**
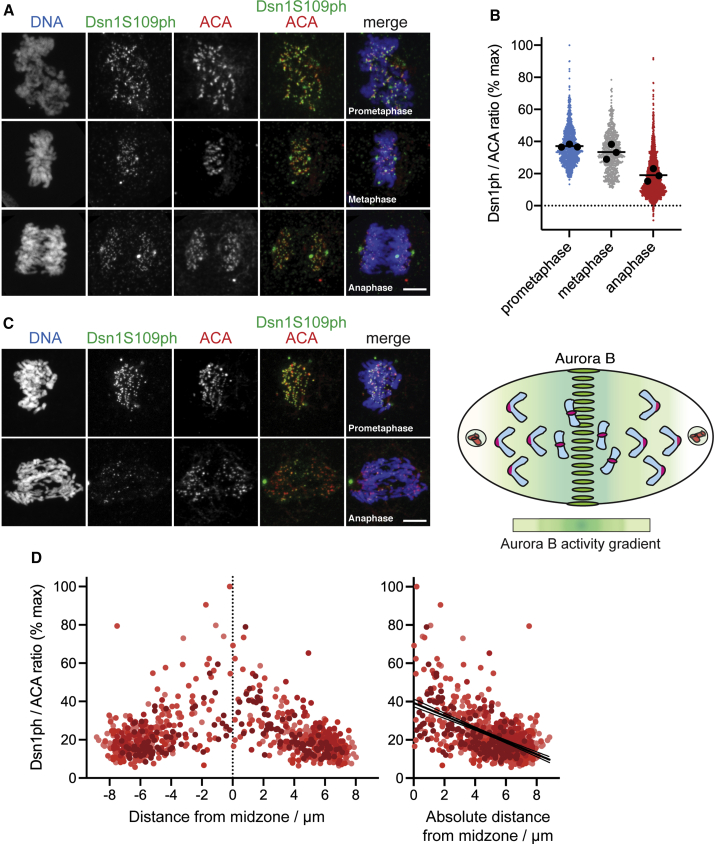
A kinetochore Aurora B substrate shows a gradient of phosphorylation in anaphase (A) HeLa cells were stained for DNA (blue), Dsn1S109ph (green), and anti-centromere autoantibody (ACA) (red). (B) Quantification of Dsn1S109ph in prometaphase (13 cells), metaphase (9 cells), and anaphase (23 cells) from three independent experiments performed as in (A). Colored dots show individual kinetochores. Black dots show the mean for each replicate; black bars show the mean of the replicates (n = 3). (C) HeLa cells treated with 10 μM MPS1-IN-1 to generate lagging chromosomes that report the gradient activity of Aurora B (see diagram) were stained as in (A). (D) Quantification of Dsn1S109ph at kinetochores as a function of distance from the midzone in 10 cells treated as in (C) shows a gradient of phosphorylation. Each dot represents an individual kinetochore, and the dots are shaded progressively according to the maximum chromosome separation distance in each cell (i.e., kinetochores in later anaphase cells are represented by lighter dots). Using linear regression, slope = −3.4 and is non-zero (p < 0.0001, F test). Confidence intervals (95%) are shown as fine lines. Scale bars: 5 μm (A and C). See also Figure S1.

When examining the Aurora B target residue S109 on the kinetochore protein Dsn1 by immunofluorescence microscopy in HeLa cells, we found that phosphorylation was observed on essentially all prometaphase kinetochores, and that this declined slightly, but clearly remained present, during metaphase (Figures 1A and 1B), as previously reported (Hadders et al., 2020; Welburn et al., 2010; Wurzenberger et al., 2012). This contrasts with phosphorylation of a number of Aurora B target sites on Knl1 and Hec1/Ndc80 that decrease substantially during metaphase (DeLuca et al., 2011; Hadders et al., 2020; Liang et al., 2020; Welburn et al., 2010). In addition, we found that Dsn1 S109 phosphorylation (Dsn1 S109ph) persisted into anaphase (Figures 1A and 1B). When HeLa cells were treated with MPS1-IN-1 to induce lagging chromosomes, a clear gradient of Dsn1 S109ph was revealed: kinetochores near the cell equator could be more strongly phosphorylated than those near the poles (Figures 1C and 1D). Linear regression confirmed that the observed gradient was significantly different from zero (p < 0.0001, F test). A similar gradient could be observed in RPE1 cells (Figures S1C and S1D). Dsn1 S109ph gradients were also observed with a second Dsn1ph S109 antibody (Figure S1A) and in the absence of MPS1-IN-1 in HeLa cells (see below) and in RPE1 cells (Figure S1E).

### Anaphase phosphorylation of Dsn1 requires the central spindle-dependent gradient of Aurora B activity

**Figure 2:**
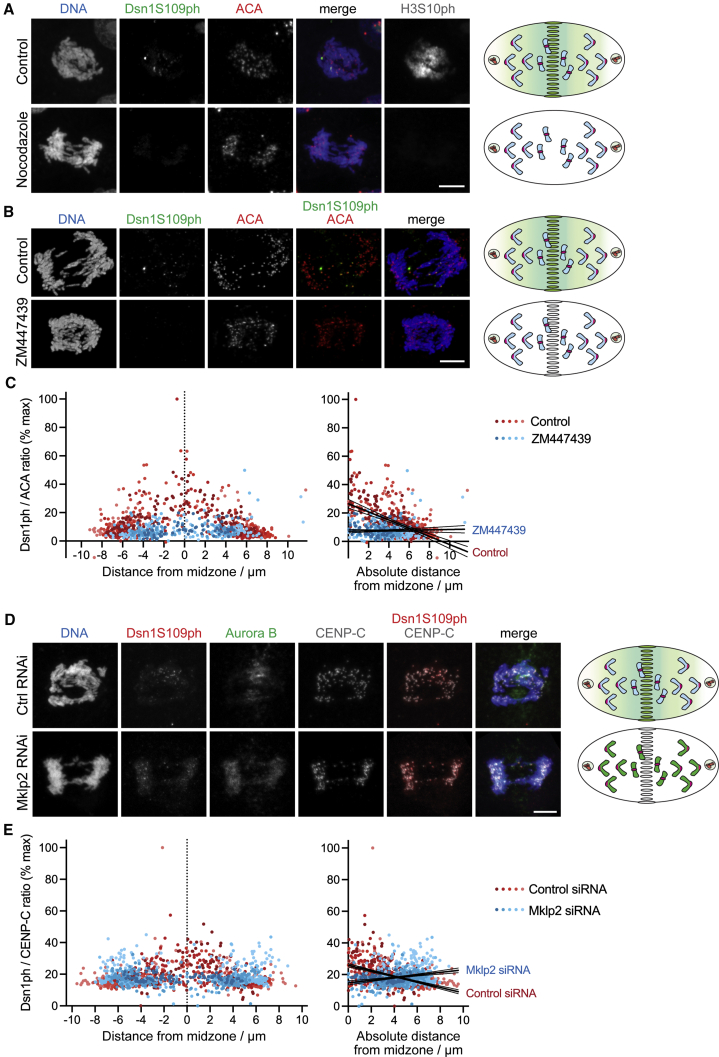
The gradient of Dsn1S109ph is dependent on central spindle Aurora B (A) Central spindle depolymerization (see diagram) eliminates the Dsn1S109ph gradient. HeLa cells were enriched in anaphases with lagging chromosomes (see STAR Methods), and nocodazole was added 5 min prior to fixation and staining for DNA (blue), Dsn1S109ph (green), ACA (red), and H3S10ph (gray). (B) Acute Aurora B inhibition eliminates the Dsn1S109ph gradient. HeLa cells were treated with MPS1-IN-1 and then with 10 μM ZM447439 for 15 min, prior to fixation and staining for DNA (blue), Dsn1S109ph (green), and ACA (red). (C) Quantification of Dsn1S109ph at individual kinetochores as a function of distance from the midzone in nine control and five ZM447439-treated cells treated as in (B). Dots are shaded as in Figure 1D. Using linear regression, for control cells, slope = −2.9 and is non-zero (p < 0.0001, F test). For ZM447439-treated cells, slope = 0.12 and is not significantly different from zero (p = 0.48). The slopes are significantly different from one another (p < 0.0001, F test). (D) Mklp2 RNAi prevents transfer of Aurora B to the central spindle (see diagram) and weakens the Dsn1S109ph gradient. Control and Mklp2-depleted HeLa cells were treated with MPS1-IN-1 and stained for DNA (blue), Dsn1S109ph (red), Aurora B (green), and CENP-C (gray). (E) Quantification of Dsn1S109ph at individual kinetochores as a function of distance from the midzone in eight control and seven Mklp2-depleted cells as in (D). Dots are shaded as in Figure 1D. Using linear regression, for control cells, slope = −1.7 and is non-zero (p < 0.0001, F test). For Mklp2-depleted cells, slope = 0.83 and also is non-zero (p < 0.0001, F test). The slopes are significantly different from one another (p < 0.0001, F test). Confidence intervals (95%) are shown as fine lines. Scale bars: 5 μm (A, B, and D). See also Figure S2.

**Figure 3:**
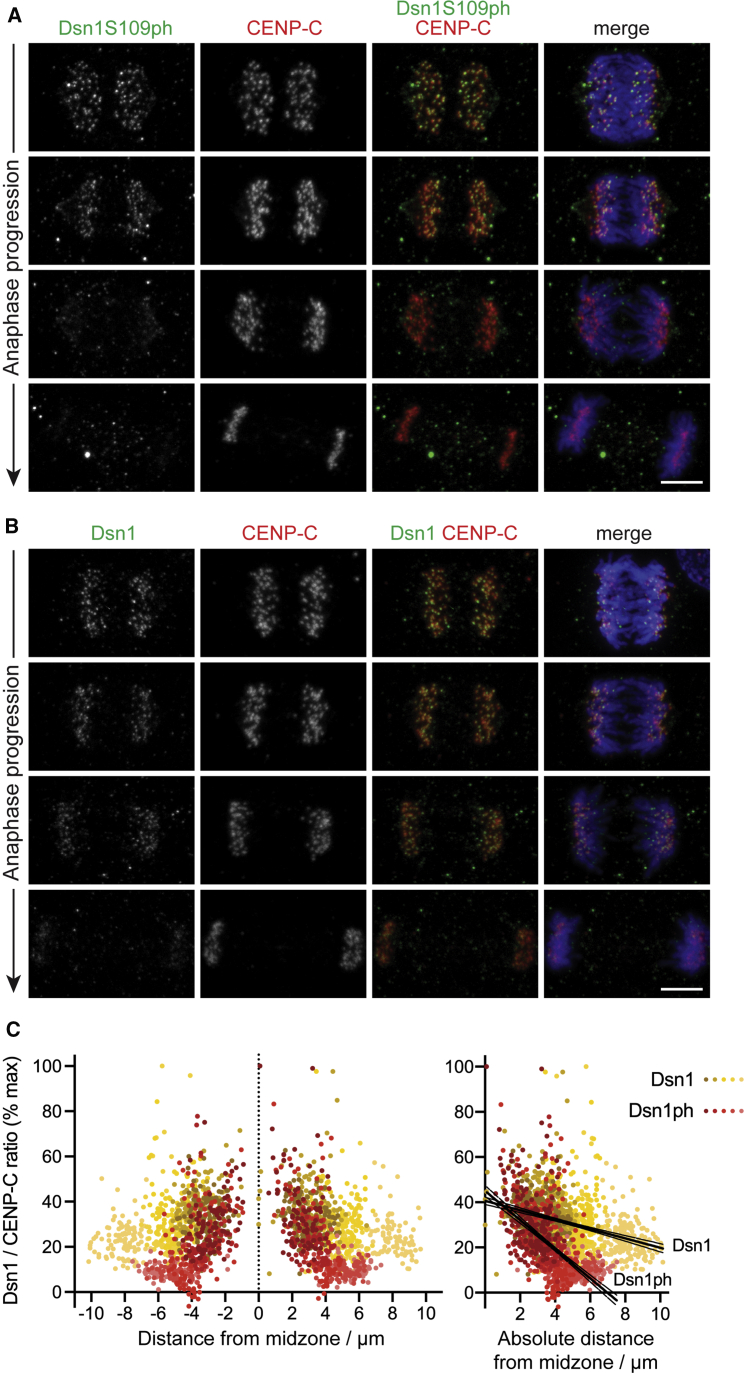
Dsn1 itself is found in a gradient at anaphase kinetochores (A) HeLa cells enriched in anaphases by thymidine release were stained for DNA (blue), Dsn1S109ph (green), and CENP-C (red). (B) HeLa cells as in (A) stained for DNA (blue), total Dsn1 (green), and CENP-C (red). (C) Quantification of Dsn1S109ph (11 cells) and total Dsn1 (15 cells) at individual kinetochores as a function of distance from the midzone. Dots are shaded as in Figure 1D. Using linear regression, for Dsn1S109ph, slope = −6.5 and is non-zero (p < 0.0001, F test). For total Dsn1, slope = −2.1 and is non-zero (p < 0.0001, F test). The slopes are significantly different from one another (p < 0.0001, F test). Confidence intervals (95%) are shown as fine lines. Scale bars: 5 μm (A and B). See also Figure S3.

Because Aurora B dissociates from chromosomes in anaphase, we sought to determine whether the observed gradient of kinetochore Dsn1 phosphorylation was due to Aurora B activity emanating from the central spindle. First, we studied the effect of acutely interfering with Aurora B location and activity in anaphase. HeLa cells were released from a nocodazole-induced mitotic arrest into medium containing MPS1-IN-1 to produce lagging chromosomes. When nocodazole was re-added to anaphase cells for 5 min to acutely depolymerize microtubules and prevent Aurora B localization to the central spindle (Figure S2A), the gradients of both H3S10ph and Dsn1 S109ph were lost (Figure 2A). Similarly, when Aurora B kinase activity was inhibited by adding the Aurora B inhibitor ZM447439 for 5 min (Figure S2B), the gradients of both H3S10ph and Dsn1 S109ph on anaphase chromosomes were again eliminated (Figure 2B). This finding was confirmed when Dsn1 S109ph was quantified in similar experiments in which ZM447439 was added for 10 min (Figure 2C) or 3 min, both with or without MPS1-IN-1 treatment (Figures S2C and S2D).

Next, we depleted Mklp2 using RNAi, which prevents the normal transfer of Aurora B to the spindle midzone and leads to its retention on chromosomes (Gruneberg et al., 2004), and examined the gradient of Dsn1 S109ph in MPS1-IN-1-treated HeLa cells. As expected, Aurora B was largely retained on chromatin and, in addition, kinetochore Dsn1 S109 no longer showed a clear gradient of phosphorylation. Instead, a relatively equal low level of phosphorylation at kinetochores across the cell was observed (Figures 2D and 2E). Similar results were obtained in the absence of MPS1-IN-1 (Figure S2E) and in RPE1 cells (Figure S2F). In a final approach, we depleted PRC1 from HeLa cells to remove central spindle Aurora B, leaving it predominantly at the equatorial cortex (Mollinari et al., 2005). In this situation, only kinetochores near Aurora B at the cell cortex were phosphorylated at Dsn1 S109 (or CENP-A S7; Figure S2G). Together, these results suggest that Dsn1 S109 phosphorylation is dependent on the gradient of Aurora B activity centered on the spindle midzone in anaphase.

### Dsn1 is found in a gradient at anaphase kinetochores

Two mechanisms each maintain up to 50% of Mis12C (containing Dsn1) at kinetochores prior to anaphase: binding to CENP-C or CENP-T (Huis In ’t Veld et al., 2016; Suzuki et al., 2015). The phosphorylation of Dsn1 at S100 and/or S109 by Aurora B is required for the stable association of Mis12C with both CENP-C and CENP-T (Akiyoshi et al., 2013; Kim and Yu, 2015; Rago et al., 2015; Walstein et al., 2021; Yang et al., 2008). Dsn1 S100/S109 phosphorylation relieves the inhibitory effect of a basic region of Dsn1 on the interaction with CENP-C and CENP-T (Dimitrova et al., 2016; Hara et al., 2018; Kim and Yu, 2015; Petrovic et al., 2016; Walstein et al., 2021). The interaction of Mis12C with CENP-T, but not with CENP-C, is additionally dependent on phosphorylation of CENP-T by Cyclin B-Cdk1 (Gascoigne and Cheeseman, 2013; Gascoigne et al., 2011; Hara et al., 2018; Huis In ’t Veld et al., 2016; Nishino et al., 2013; Walstein et al., 2021). To determine whether, like Dsn1 S109ph, total Dsn1 is also found in a gradient, we compared the distribution of phosphorylated and total Dsn1 in HeLa cells undergoing normal anaphase. As expected (Gascoigne and Cheeseman, 2013; Kim and Yu, 2015; Rago et al., 2015; Yang et al., 2008), Dsn1 persisted at kinetochores in anaphase, although it declined as anaphase progressed (Figures 3A and 3B). In anaphases of MPS1-IN-1-treated cells, a gradient of Dsn1 localization intensity could also be observed, including in single cells, suggesting that Dsn1 localization to kinetochores is spatially regulated (Figures S3A and S3B). Interestingly, however, the gradient of total Dsn1 at anaphase kinetochores was shallower than that observed for Dsn1 S109ph, meaning that Dsn1 appeared to persist at poleward kinetochores for longer than Dsn1 S109ph (Figure 3C). This suggests that there is a temporal delay in the release of Dsn1 from kinetochores following its dephosphorylation and/or that there is a population of Dsn1 that can localize to kinetochores until late anaphase independently of Dsn1 S109 phosphorylation.

### Phosphorylation of Dsn1 modulates Dsn1 localization at kinetochores in anaphase

If the localization of Dsn1 to kinetochores in anaphase depends in part on its phosphorylation, as in early mitosis, then the normal distribution of Dsn1 in anaphase should require Aurora B activity. Short-term inhibition of Aurora B (5–10 min), in either the presence or absence of MPS1-IN-1, had a minor effect on total Dsn1 signal at anaphase kinetochores in immunofluorescence experiments (Figure S3C and S3D), suggesting a subtle effect of Aurora B inhibition on Dsn1. Consistent with this, short-term inhibition of Aurora B was insufficient to substantially remove Dsn1 from kinetochores in prometaphase, although Dsn1 S109ph and H3S10ph were reduced (Figure S3E).

**Figure 4:**
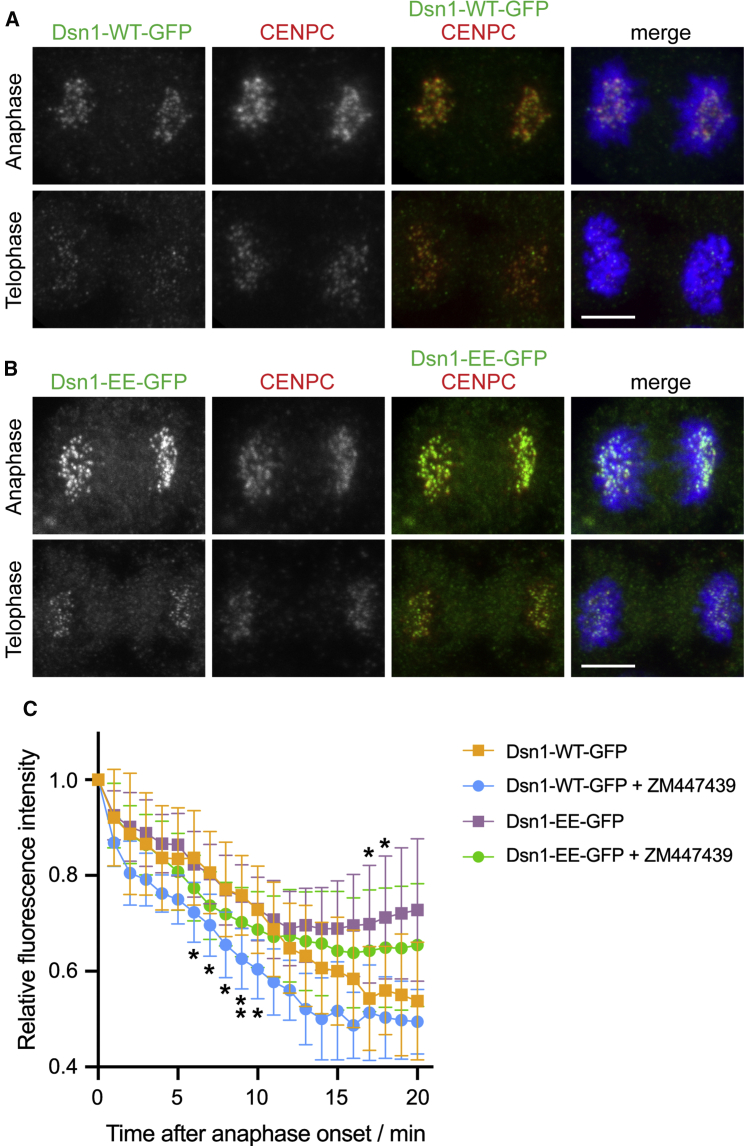
Aurora B phosphorylation modulates Dsn1 localization at anaphase kinetochores (A) HeLa cells stably expressing Dsn1-WT-GFP were stained to visualize DNA (blue), GFP (green), and CENP-C (red). (B) HeLa cells stably expressing Dsn1-EE-GFP were stained as in (A). (C) Quantification of the kinetochore fluorescence of Dsn1-WT-GFP and Dsn1-EE-GFP in living HeLa cells imaged at 1-min intervals. Values were normalized to 1 at anaphase onset. Where indicated, cells were treated between 0 and 10 min prior to anaphase onset with 4 or 10 μM ZM447439. For Dsn1-WT-GFP ± ZM447439, n = 8; for Dsn1-EE-GFP ± ZM447439, n = 12 and 10. Data are represented as mean ± SD. ^∗^p < 0.05, ^∗∗^p = 0.0085, by two-way ANOVA followed by Dunnett’s multiple comparisons test. Scale bars: 5 μm (A and B). See also Figure S4 and Videos S1, S2, S3, and S4.

As an alternative and more tractable system to examine Dsn1 localization, we turned to fluorescent Dsn1 fusion proteins. Specifically, we compared the behavior of Dsn1-WT (wild-type)-GFP and a mutant in which S100 and S109 were replaced with phospho-mimetic glutamate residues (Dsn1-EE-GFP) (Kim and Yu, 2015). As seen for endogenous Dsn1 (Figures 3A and 3B), Dsn1-WT-GFP was detected on kinetochores during anaphase but was reduced in telophase (Figure 4A). Dsn1-EE-GFP was also present on kinetochores during anaphase (Figure 4B), but it persisted into telophase more clearly than Dsn1-WT-GFP, as previously reported (Kim and Yu, 2015). Because Dsn1-GFP expression levels varied from cell to cell, it was not straightforward to use fluorescence microscopy of fixed cells to compare its localization at different stages of anaphase. Therefore, we used live cell imaging to follow the localization of Dsn1-GFP at kinetochores during anaphase in individual cells (Figure 4C). This confirmed that Dsn1-WT-GFP localization at kinetochores declined as chromosomes moved poleward in anaphase (Video S1), as observed for endogenous Dsn1. Dsn1-EE-GFP showed a similar decline at kinetochores in anaphase (Video S2) but was retained in telophase to a greater extent than Dsn1-WT-GFP. Short-term treatment with Aurora B inhibitor modestly, but significantly, increased the rate of Dsn1-WT-GFP dissociation from kinetochores in anaphase (Video S3), while Dsn1-EE-GFP was less affected (Video S4). In addition, the persistent localization of Dsn1-EE-GFP on telophase chromosomes was not greatly affected by Aurora B inhibitor (Figure 4C).

Video S1. Dsn1-WT-GFP (left panel) and DNA labeled with SiR-DNA (right panel) were imaged in a living HeLa cell at 1-min intervals, related to Figure 4 Deconvolved images are shown.

Video S2. Dsn1-WT-GFP (left panel) and DNA labeled with SiR-DNA (right panel) were imaged in a living HeLa cell at 1-min intervals, related to Figure 4 4 μM ZM447439 was added approximately 2 min prior to time = 0 min. Deconvolved images are shown.

Video S3. Dsn1-EE-GFP (left panel) and DNA labeled with SiR-DNA (right panel) were imaged in a living HeLa cell at 1-min intervals, related to Figure 4 Deconvolved images are shown.

Video S4. Dsn1-EE-GFP (left panel) and DNA labeled with SiR-DNA (right panel) were imaged in a living HeLa cell at 1-min intervals, related to Figure 4 4 μM ZM447439 was added approximately 5 min prior to time = 0 min. Deconvolved images are shown.

To explore these results further, we generated a simple mathematical model for the dissociation of Mis12C from CENP-C and CENP-T at anaphase kinetochores. We made the simplifying assumption that the dissociation can be described as a two-phase exponential decay. We made the following additional assumptions. First, at the start of anaphase, 50% of Mis12C is associated with CENP-T, regulated by both Aurora B and Cyclin B-Cdk1, and 50% is associated with CENP-C in an Aurora B-dependent manner (Huis In ’t Veld et al., 2016; Suzuki et al., 2015; Walstein et al., 2021). Second, the dissociation of Cdk1-dependent Mis12C is likely to be faster than that of Aurora B-dependent Mis12C. Indeed, Cyclin B1 destruction reaches completion in early anaphase (Afonso et al., 2019; Clute and Pines, 1999), and many Cdk1-generated phosphosites are removed rapidly in anaphase (Vandré and Borisy, 1989), including in the N-terminal region of CENP-T (Gascoigne et al., 2011). Third, the affinity of Aurora B-phosphorylated Mis12C for CENP-C and Cdk1-phosphorylated CENP-T is similar, as reported previously (Walstein et al., 2021). The model can recapitulate key features of the results, such as the similar kinetics of Dsn1-WT and Dsn1-EE dissociation from kinetochores in early anaphase (likely largely because of loss of Cdk1-dependent CENP-T binding), and the failure of approximately 50% of Dsn1 to dissociate from kinetochores in telophase when the half-life of Aurora B-dependent Mis12C is increased (Figure S4A), as seen for the Dsn1-EE mutant (Figure 4C). Fitting the model to the data in Figure 4C using non-linear regression (Figure S4B) suggested an approximate half-life for Cdk1-dependent Mis12C in anaphase of 5 min. The half-life of Aurora B-dependent Mis12C was approximately 17 min, declining to 5 min when Aurora B was inhibited (p = 0.011, extra sum-of-squares F test).

These results suggested that phosphorylation of S100 and/or S109 by Aurora B is required for the normal maintenance of Dsn1 localization at kinetochores in anaphase, and that dephosphorylation is required for the complete release of Dsn1 from kinetochores in telophase.

### Dsn1 phosphorylation by Aurora B modulates kinetochore disassembly in late anaphase

**Figure 5:**
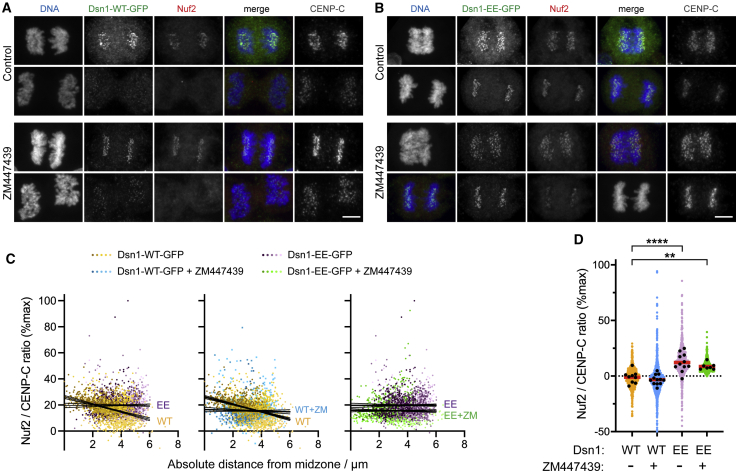
Dsn1 phosphorylation by Aurora B modulates kinetochore disassembly in late anaphase (A) HeLa cells expressing Dsn1-WT-GFP were stained for DNA (blue), GFP (green), Nuf2 (red), and CENP-C (gray). Where indicated, cells were treated with 10 μM ZM447439 for 15 min prior to fixation. (B) HeLa cells expressing Dsn1-EE-GFP were treated and stained as in (A). (C) In anaphase cells treated as in (A) and (B), Nuf2 at individual kinetochores was quantified as a function of distance from the midzone. Dots are shaded as in Figure 1D. Using linear regression, for 17 Dsn1-WT-GFP cells, slope = −2.9 and is non-zero (p < 0.0001, F test). For 17 ZM447439-treated Dsn1-WT-GFP cells, 20 Dsn1-EE-GFP cells, and 16 ZM447439-treated Dsn1-EE-GFP cells, the slopes are −0.3, 0.03, and −0.1, respectively, which are not significantly different from zero (p = 0.29, 0.86, and 0.60). Comparing Nuf2 in Dsn1-WT-GFP cells with and without ZM447439, and comparing Nuf2 in Dsn1-WT-GFP with Dsn1-EE-GFP cells, the slopes are significantly different from one another (p < 0.0001, F test). Confidence intervals (95%) are shown as fine lines. (D) In telophase cells treated as in (A) and (B), Nuf2 was quantified at kinetochores. Colored symbols show the results for individual kinetochores, black dots show the means for individual cells, and red bars show the means of these means. For Dsn1-WT-GFP cells ± ZM447439, n = 9 and 12; for Dsn1-EE-GFP cells ± ZM447439, n = 11 and 7. ^∗∗^Adjusted p = 0.0054; ^∗∗∗∗^adjusted p < 0.0001, by one-way ANOVA followed by Dunnett’s multiple comparisons test. Scale bars: 5 μm (A and B).

In current models, the recruitment of other KMN components to kinetochores in early mitosis is partly dependent on the binding of Mis12C to CCAN proteins (Musacchio and Desai, 2017). To determine whether Dsn1 phosphorylation modulates the localization of additional kinetochore components during mitotic exit, we examined Nuf2, a component of the Ndc80 complex (Ndc80C). Like Dsn1 itself, Nuf2 was lost from kinetochores in Dsn1-WT-GFP-expressing HeLa cells as anaphase progressed (Figures 5A and 5C), and kinetochore localization was low in telophase cells (Figure 5D). However, in cells expressing Dsn1-EE-GFP, the decline in kinetochore Nuf2 was less clear (Figures 5B and 5C), and it remained detectable at kinetochores into telophase (p < 0.0001, one-way ANOVA followed by Bonferroni’s multiple comparison test; Figure 5D). Inhibition of Aurora B in cells expressing Dsn1-WT-GFP compromised the Nuf2 gradient in anaphase (Figure 5C), although we noted that Aurora B inhibition also had some effect in cells expressing Dsn1-EE-GFP (Figure 5C), suggesting an additional Aurora B contribution independent of Dsn1 S100/S109 phosphorylation. The localization of Nuf2 to telophase kinetochores in Dsn1-EE-GFP-expressing cells was largely resistant to Aurora B inhibitor treatment (Figure 5D), suggesting that dephosphorylation of Dsn1 S100/S109 is involved in the disassembly of the kinetochore as cells enter telophase.

### Aurora B maintains kinetochore function in anaphase

**Figure 6:**
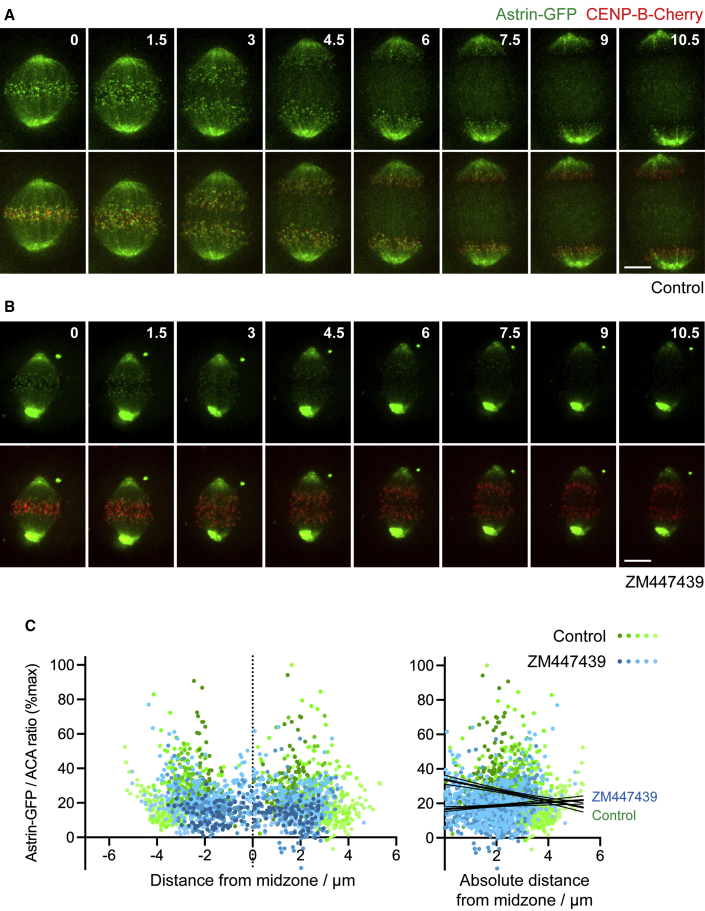
Astrin localization is influenced by Aurora B in anaphase (A) A HeLa cell stably expressing Astrin-GFP (green) and CENP-B-Cherry (red) was imaged every 30 s by iSIM super-resolution microscopy. (B) A cell as in (A) was treated with 5 μM ZM447439 immediately prior to imaging, which began at time = 0. (C) HeLa cells expressing Astrin-GFP were treated with 5 μM ZM447439 or control for 10 min, then cold treated to depolymerize labile microtubules, fixed, and stained for DNA, GFP, and ACA. Astrin-GFP at individual kinetochores was quantified as a function of distance from the midzone. Dots are shaded as in Figure 1D. Using linear regression, for 13 control cells, slope = −3.0, which is non-zero (p < 0.0001, F test), and for 12 ZM447439-treated cells, slope = 0.99, which is non-zero (p = 0.0027, F test). The slopes are significantly different from one another (p < 0.0001, F test). Confidence intervals (95%) are shown as fine lines. Scale bars: 5 μm (A and B). See also Figure S5 and Videos S5 and S6.

To monitor kinetochore-microtubule attachments in anaphase, we turned to Astrin, a marker of stable kinetochore-microtubule attachments (Mack and Compton, 2001). The localization of Astrin to kinetochores is modulated by KMN components (Kern et al., 2017; Manning et al., 2010; Wang et al., 2012). Astrin becomes localized at kinetochores as microtubules form end-on attachments in prometaphase, and it clearly decorates kinetochores in metaphase and into anaphase (Du et al., 2008; Dunsch et al., 2011; Shrestha et al., 2017). Imaging of HeLa cells expressing Astrin-GFP (Dunsch et al., 2011) and CENP-B-Cherry showed that Astrin-GFP remained detectable at kinetochores during anaphase until the chromosomes are in close proximity to the spindle poles, when Astrin appeared to be fully released from kinetochores (Figure 6A; Video S5). In cells treated with Aurora B inhibitor, chromosomes moved more slowly away from the midzone (Afonso et al., 2014; Wurzenberger et al., 2012) and, in several cases, did not approach as closely to the spindle poles. In these cells, Astrin was lost from kinetochores closer to the midzone than in control cells, coinciding with the termination of chromosome movement prior to reaching the spindle poles (Figure 6B; Video S6). Furthermore, in untreated cells with spontaneously lagging or bridging chromosomes, Astrin-GFP appeared to persist on kinetochores closer to the midzone (Figure S5A; Video S7).

Video S5. HeLa cell expressing Astrin-EGFP (green) and CENP-B-Cherry (red) was imaged every 30 s, related to Figure 6A Deconvolved images are shown.

Video S6. A HeLa cell expressing Astrin-EGFP (green) and CENP-B-Cherry (red) was imaged every 30 s, related to Figure 6B 5 μM ZM447439 was added approximately 2 min prior to time = 0 min. Deconvolved images are shown.

Video S7. A HeLa cell expressing Astrin-EGFP (green) and CENP-B-Cherry (red) with lagging kinetochores was imaged every 30 s, related to Figure S5A Deconvolved images are shown.

In these live imaging experiments, the presence of Astrin on microtubules near the spindle poles (Mack and Compton, 2001) introduced ambiguity into the timing of Astrin release from kinetochores. As an alternative method, we turned to immunofluorescence of cold-treated anaphase cells. Cold treatment favors disassembly of non-kinetochore microtubules in anaphase (Brinkley and Cartwright, 1975), and we noticed that it reduced the intensity of Astrin on the spindle, allowing clearer visualization of kinetochore Astrin. Cold treatment may also reveal the influence of Aurora B on the stability of kinetochore attachments by favoring the disassembly of unstable kinetochore microtubules. Using this approach, we confirmed that Astrin-GFP levels on anaphase kinetochores were decreased by Aurora B inhibitor treatment (Figure 6C). Similar results were obtained in MPS1-IN-1-treated cells (Figures S5B and S5C). The effect of Mklp2 depletion on Astrin was harder to determine because, in this condition, active Aurora B is retained on chromosomes in anaphase rather than its activity being eliminated (see above). However, we found that the gradient of endogenous Astrin was compromised when Aurora B was inhibited in cells expressing Dsn1-WT-GFP, but less so in cells expressing Dsn1-EE-GFP (Figure S5D). Clearly Aurora B influences the function of other spindle components (Afonso et al., 2017), but these results are consistent with the idea that Aurora B activity is required to maintain the stability of kinetochores as they move away from the midzone in anaphase.

**Figure 7:**
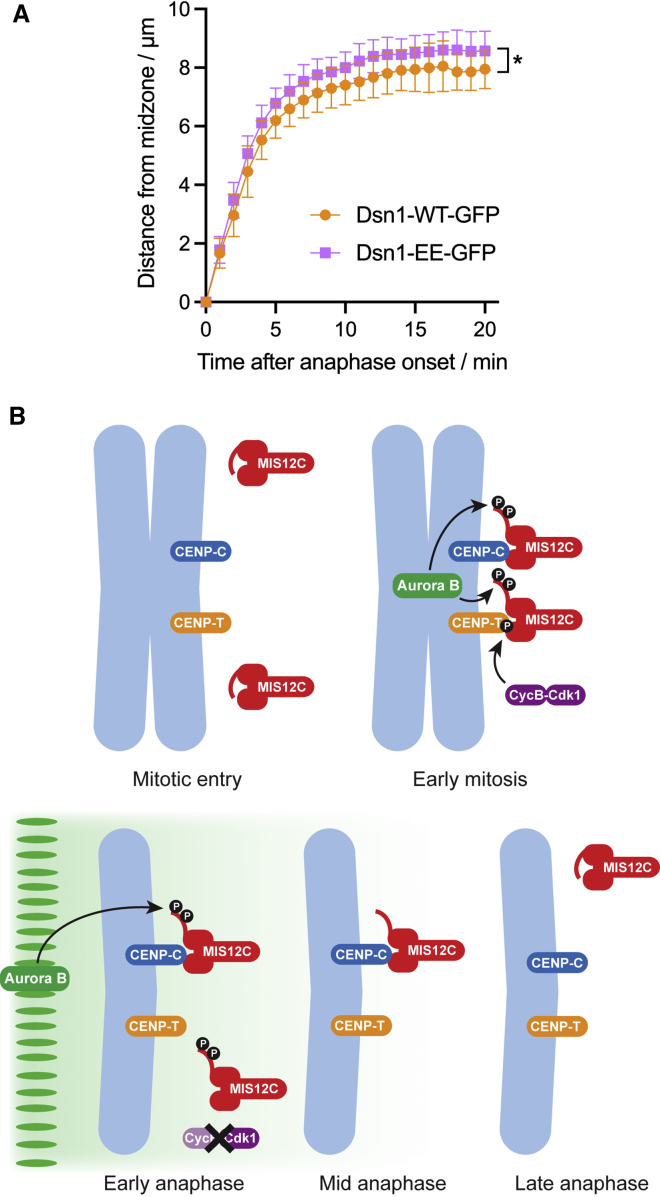
Mis12C regulation of anaphase kinetochores (A) Chromosome movements in living HeLa cells expressing Dsn1-WT-GFP or Dsn1-EE-GFP imaged at 1-min intervals. For Dsn1-WT-GFP, n = 10; and for Dsn1-EE-GFP, n = 11. Data are represented as mean ± SD. ^∗^p = 0.028 by two-way ANOVA. (B) Model. In early mitosis, phosphorylation at S100/S109 by Aurora B (green) displaces the basic region of Dsn1 and allows Mis12C (red) binding to CENP-C (blue) and CENP-T (orange). Phosphorylation of CENP-T by Cyclin B-Cdk1 (purple) is also required for Mis12C to bind CENP-T. In early anaphase, Cyclin B degradation and loss of Cdk1-dependent CENP-T phosphorylation releases Mis12C from CENP-T, while Aurora B gradient activity can prolong phosphorylation of Dsn1 S100/S109, allowing Mis12C retention on CENP-C. As chromosomes move away from the central spindle, declining Aurora B activity allows dephosphorylation of Dsn1 S100/S109, although full release of Mis12C is delayed because phosphorylation is not directly required for CENP-C binding. By late anaphase, Dsn1 returns to its autoinhibited conformation, and all Mis12C is released.

Finally, to determine if phosphorylation of Aurora B target sites on Dsn1 influences chromosome movements in anaphase, we measured the rate at which chromosomes separated in HeLa cells expressing Dsn1-WT-GFP or Dsn1-EE-GFP. Although the effect was modest, chromosome movement slowed down less quickly toward the end of anaphase in cells expressing Dsn1-EE-GFP (p = 0.028 by two-way ANOVA; Figure 7A), consistent with the idea that Dsn1 S100/S109 phosphorylation by Aurora B contributes to the stability of kinetochores to promote chromosome movement in anaphase.

## Discussion

The use of artificial centromere-targeted substrates previously revealed a gradient of Aurora B activity centered on the spindle midzone in anaphase (Fuller et al., 2008). Here we show that phosphorylation of a natural kinetochore substrate (Dsn1 S109) is sensitive to its distance from midzone Aurora B, suggesting that the Aurora B gradient modulates the activity of kinetochores themselves during anaphase. Prior to anaphase, phosphorylation of Dsn1 S100 and S109 by centromeric Aurora B promotes the association of Mis12C with its centromere receptors, CENP-C and CENP-T (Akiyoshi et al., 2013; Bonner et al., 2019; Dimitrova et al., 2016; Hara et al., 2018; Kim and Yu, 2015; Petrovic et al., 2016; Rago et al., 2015; Walstein et al., 2021; Yang et al., 2008). Here we find that Aurora B also regulates Dsn1 localization at kinetochores in anaphase. Specifically, midzone Aurora B-mediated phosphorylation of S100/S109 reduces the rate at which Dsn1 is lost from kinetochores as anaphase progresses. Loss of Aurora B activity also leads to premature loss of Astrin, a marker of stable microtubule attachments, from kinetochores. Therefore, the anaphase gradient of Aurora B serves to prolong the stability of kinetochores at a time when the loss of centromeric Aurora B might otherwise lead to premature kinetochore disassembly.

It is remarkable that structural components of the kinetochore such as Dsn1 appear to be shed as chromosomes are moving to the poles in anaphase, but this phenomenon has been reported for a number of KMN proteins (Gascoigne and Cheeseman, 2013; McAinsh et al., 2006), and microtubule attachments appear relatively insensitive to the amount of Mis12C or functional Ndc80/Hec1 at kinetochores (Kukreja et al., 2020; Zaytsev et al., 2014). Nevertheless, it is possible that shedding represents continued remodeling of the kinetochore to provide dynamics optimal for anaphase chromosome movements. Indeed, it is notable that chromosomes slow down as anaphase progresses (Zhai et al., 1995), perhaps reflecting changes in kinetochore function and microtubule dynamics. In fact, we find that the timely loss of Aurora B-mediated phosphorylation on Dsn1 S100/S109 is required for the normal dissociation of KMN proteins from kinetochores at the end of anaphase, and that expression of Dsn1-EE causes chromosomes to sustain their movement toward the poles for longer than in control cells.

It is also striking that the spatial gradient of total Dsn1 levels at anaphase kinetochores is shallower than that of Dsn1 S109ph, and that Dsn1 appears to remain at kinetochores for a time even when Dsn1 S109 is no longer phosphorylated, and when any possible Cdk1-dependent localization of Mis12C is expected to be low (see below). This may be because of a previously unexplained feature of Dsn1 regulation: that S100/S109 phosphorylation does not directly enhance binding to CENP-C (and CENP-T), but rather it displaces an autoinhibitory loop of Dsn1 to reveal the CENP-C binding region (Dimitrova et al., 2016; Hara et al., 2018; Kim and Yu, 2015; Petrovic et al., 2016; Walstein et al., 2021). This mechanism means that the initial stable binding of Mis12C to kinetochores is highly dependent on phosphorylation of Dsn1 S100/S109 and requires high local Aurora B activity (Bonner et al., 2019), but that dephosphorylation does not necessarily lead to the immediate release of Dsn1. This would allow Mis12C to persist at kinetochores as Aurora B activity declines to maintain kinetochore function (Figure 7B), a model supported by the reported high affinity of Mis12C binding to CENP-C (Petrovic et al., 2016). We speculate that this property of autoinhibitory mechanisms may be important in this and other cellular systems.

In early mitosis, Aurora B contributes to both the assembly of kinetochores (e.g., through Dsn1 S100/S109 phosphorylation) and the destabilization of kinetochore-microtubule interactions to facilitate error correction (e.g., through phosphorylation of Ndc80/Hec1). This raises the question of whether the Aurora B gradient is able to destabilize microtubule attachments in anaphase, such as on the midzone-proximal regions of kinetochores of merotelic lagging chromosomes. Although this model has some appeal, and we cannot entirely rule it out because of technical issues, we have been unable to demonstrate phosphorylation of Ndc80/Hec1 on Aurora target sites such as S44 and S55 in early anaphase (data not shown). Aurora B-mediated phosphorylation of Ndc80/Hec1 declines more strongly in metaphase than that of Dsn1 (DeLuca et al., 2011; Hadders et al., 2020; Liang et al., 2020; Welburn et al., 2010; Wurzenberger et al., 2012), suggesting that Dsn1 phosphorylation requires lower Aurora B activity levels than that of Ndc80/Hec1. Indeed, Dsn1 S100/S109 appear to be particularly good substrates for Aurora B *in vitro*, particularly once autoinhibition has been relieved (Bonner et al., 2019; Zhou et al., 2017). It appears likely that the level of Aurora B gradient activity experienced by kinetochores in early anaphase is sufficient to maintain Dsn1 phosphorylation to stabilize kinetochores but is insufficient to destabilize kinetochore-microtubule interactions. This may also help explain why Aurora B inhibition does not increase Astrin localization to anaphase kinetochores as it can in prometaphase (Manning et al., 2010; Schmidt et al., 2010). At least in part, inhibiting Aurora B appears to increase Astrin localization to prometaphase kinetochores because Astrin binds preferentially to stably attached kinetochores (Friese et al., 2016; Schmidt et al., 2010). By contrast, in anaphase, the main effect of Aurora B inhibition is to destabilize kinetochore structure, an outcome that may be particularly evident when Cdk1 activity is low. Other substrates that are sensitive to the Aurora B gradient in anaphase include histone H3S10 (Afonso et al., 2014; Fuller et al., 2008) and CENP-AS7 (this study), and we note that phosphorylation of Dsn1 may not be the only mechanism by which Aurora B influences kinetochore function in anaphase.

In previous work, it was found that the depletion of phosphatase regulators such as Sds22 and Repo-Man caused increased phosphorylation of Dsn1 S100 and transient pauses in anaphase chromosome migration (Wurzenberger et al., 2012). At that time, it was not known that Dsn1 S100ph is involved in stabilizing kinetochores rather than in destabilizing microtubule attachments. In light of our study, it is perhaps more likely that failure to dephosphorylate Dsn1 compromises necessary changes in the structural properties of the kinetochore itself and so indirectly alters the dynamics required for chromosome movement. Alternatively, phosphatase depletion might allow other Aurora B targets such as Ndc80/Hec1 to be over-phosphorylated to destabilize attachments. Whether the phosphorylation of Aurora B substrates can reach levels high enough to destabilize microtubule attachments on lagging chromosomes in otherwise normal cells remains an open question.

As well as Aurora B, Cyclin B-Cdk1 plays a vital role in building kinetochores (Gascoigne and Cheeseman, 2013; Gascoigne et al., 2011; Hara et al., 2018; Huis In ’t Veld et al., 2016; Nishino et al., 2013; Walstein et al., 2021), and our study is consistent with loss of Cdk1 activity also playing an important role in regulating kinetochore structure in anaphase (Figure 7B). Indeed, before anaphase, up to 50% of Dsn1 molecules require phosphorylation of CENP-T by Cdk1 to be recruited to kinetochores (Huis In ’t Veld et al., 2016; Suzuki et al., 2015). It is clear that Dsn1 with phospho-mimetic residues at S100 and S109 is removed from kinetochores in early anaphase with kinetics similar to WT Dsn1 (Figure 4C). Our two-phase decay model for loss of Dsn1 from kinetochores suggests that the Cdk1-mediated CENP-T-dependent population of Dsn1 dissociates with a half-life of approximately 5 min, consistent with the rapid dephosphorylation of many Cdk1-dependent sites in anaphase (Gascoigne et al., 2011; Vandré and Borisy, 1989). Non-degradable Cyclin B causes aberrant anaphase chromosome movements (Parry et al., 2003; Su et al., 2016), consistent with the need for timely dephosphorylation of substrates such as CENP-T. On the other hand, short-lived residual Cdk1 activity in early anaphase (Afonso et al., 2019) may sustain Dsn1 at kinetochores for a short time, influenced also by the rate of CENP-T dephosphorylation (Gascoigne and Cheeseman, 2013). Together, Cdk1 and Aurora B co-operate to regulate Dsn1 localization and kinetochore structure in anaphase (Figure 7B).

In summary, we have shown that phosphorylation driven by the Aurora B gradient helps sustain kinetochore structure over the time and distance necessary for normal anaphase chromosome segregation and regulates kinetochore disassembly as cells enter telophase. This spatial regulation of kinetochore phosphorylation may also allow kinetochore stability on lagging chromosomes to be maintained to facilitate their movement to the poles in anaphase (Orr et al., 2021 [this issue of *Cell Reports*]).

## STAR★Methods

### Key resources table

**Table:** 

REAGENT or RESOURCE	SOURCE	IDENTIFIER
**Antibodies**		

Rabbit polyclonal anti-Aurora B (1:1000)	Abcam	Cat#ab2254; RRID:AB_302923
Rabbit polyclonal anti-Nuf2 (1:500)	Abcam	Cat#ab122962; RRID:AB_10902068
Rabbit polyclonal anti-Dsn1 (1:1000)	Iain Cheeseman; Welburn et al., 2010	19.2B
Rabbit polyclonal anti-Dsn1S109ph (1:1000)	Iain Cheeseman; Welburn et al., 2010	19.2A
Rabbit polyclonal anti-Dsn1S109ph (1:1000)	Iain Cheeseman; Welburn et al., 2010	20.2A
Rabbit polyclonal anti-CENP-AS7ph (1:500)	Upstate/Millipore	Cat#07-232; RRID:AB_310845
Rabbit polyclonal anti-Astrin (1:1000)	Duane Compton; Mack and Compton, 2001	N terminus 1-609
Mouse monoclonal anti-Aurora B, clone AIM-1 (1:100)	BD Bioscience	Cat#611082; RRID:AB_2227708
Mouse monoclonal anti-H3S10ph, clone 6G3 (1:500)	Cell Signaling Technology	Cat#9706; RRID:AB_331748
Guinea pig polyclonal anti-CENP-C (1:1000)	MBL International	Cat#PD030; RRID:AB_10693556
Chicken polyclonal anti-GFP (1:1000)	Applied Biological Materials	Cat#G160
Human anti-centromere autoantibody (ACA; 1:1000)	Immunovision/Erba Diagnostics	Cat#HCT-0100; RRID:AB_2744669
Donkey anti-rabbit IgG Alexa Fluor Plus 488	ThermoFisher	Cat#A-32790; RRID:AB_2762833
Donkey anti-mouse IgG Alexa Fluor Plus 488	ThermoFisher	Cat#A-32766; RRID:AB_2762823
Donkey anti-rabbit IgG Alexa Fluor Plus 594	ThermoFisher	Cat#A-32754; RRID:AB_2762827
Donkey anti-mouse IgG Alexa Fluor Plus 594	ThermoFisher	Cat#A-32744; RRID:AB_2762826
Goat anti-guinea pig IgG Alexa Fluor 647	ThermoFisher	Cat#A-21450; RRID:AB_141882
Goat anti-human IgG Alexa Fluor 647	ThermoFisher	Cat#A-21445; RRID:AB_2535862
Goat anti-chicken IgY Alexa Fluor 488	ThermoFisher	Cat#A-21449; RRID:AB_2535866

**Biological samples**		

Bovine Serum Albumin	Rockland	Cat#BSA-50
Fetal Bovine Serum	LabTech	Cat#FCS-SA/500-70428

**Chemicals, peptides, and recombinant proteins**		

Doxycycline	ThermoFisher	Cat#26531
G418	ThermoFisher	Cat#10131035
Hygromycin B	ThermoFisher	Cat#10687010
MPS1-IN-1	MedChem Express	Cat#HY-13298; CAS 1125593-20-5
Nocodazole	Sigma-Aldrich	Cat#M1404; CAS 31430-18-9
Puromycin	ThermoFisher	Cat#12122530
SiR-DNA	Spirochrome	Cat#SC007
Thymidine	Sigma-Aldrich	Cat#T1895
ZM447439	Tocris	Cat#2458; CAS 331771-20-1
ProLong Diamond with DAPI	ThermoFisher	Cat#P36962
ProLong Glass with NucBlue	ThermoFisher	Cat#P36981
Methanol	ThermoFisher	Cat#M/4000/17
Paraformaldehyde	ThermoFisher	Cat#28908
Dulbecco’s Modified Eagle’s Medium (DMEM)	Sigma-Aldrich	Cat#D5796
Fluorobrite DMEM medium	ThermoFisher	Cat#A1896701
DMEM/F12 medium	Sigma-Aldrich	Cat#D6421
L-15 medium	ThermoFisher	Cat#11415064
Phosphate-Buffered Saline (PBS)	Sigma Aldrich	Cat#D8537
Penicillin-Streptomycin	Sigma Aldrich	Cat#P4333

**Critical commercial assays**		

HiPerfect transfection reagent	QIAGEN	Cat#301704
X-TremeGENE 9	Roche	Cat#6365787001

**Experimental models: Cell lines**		

HeLa Kyoto	EMBL Heidelberg	RRID: CVCL_1922
HeLa-TetOn cells expressing Dsn1-EGFP and Dsn1-EE-EGFP	Soonjoung Kim and Hongtao Yu; Kim and Yu, 2015	N/A
HeLa expressing Astrin-EGFP	Ulrike Gruneberg; Dunsch et al., 2011	N/A
hTERT-RPE1	ATCC	Cat#CRL-4000; RRID: CVCL_4388

**Oligonucleotides**		

siRNA Mklp2, 5′-CCACCUAUGUAAUCUCAUGTT-3′	Integrated DNA Technologies	Custom
siRNA PRC1, ON-TARGETplus siRNA SMARTpool	Dharmacon	Cat#L-019491-00-0005
AllStars negative control siRNA	QIAGEN	Cat#1027281

**Recombinant DNA**		

pCENP-B-Cherry	Michael Lampson; Liu et al., 2010	Addgene #45219
pPGKpuro	Rudolf Jaenisch; Tucker et al., 1996	Addgene #11349

**Software and algorithms**		

Fiji 2.1.0 software	Schindelin et al., 2012	https://fiji.sc; RRID:SCR_002285
Imaris x64 9.6.0 software	Oxford Instruments	https://imaris.oxinst.com; RRID:SCR_007370
Huygens Software 20.04	Scientific Volume Imaging	https://svi.nl/Huygens-Software; RRID:SCR_014237
Leica Application Suite X v3 software	Leica Microsystems	https://www.leica-microsystems.com; RRID:SCR_013673
Nikon Elements 5.22 software	Nikon Corporation	https://www.microscope.healthcare.nikon.com; RRID:SCR_014329
Zeiss ZEN 2.3 software	Carl Zeiss AG	https://www.zeiss.com/microscopy/int/home.html; RRID:SCR_013672
Prism 9.0.1 software	GraphPad	https://www.graphpad.com/scientific-software/prism/; RRID:SCR_002798

**Other**		

Poly-L-lysine-coated coverslips	Corning BioCoat	Cat#354085
FluoroDish	WPI Inc.	Cat#FD35-100
SP8 confocal microscope	Leica Microsystems	https://www.leica-microsystems.com
A1R confocal microscope	Nikon Corporation	https://www.microscope.healthcare.nikon.com
AxioImager microscope	Carl Zeiss AG	https://www.zeiss.com/microscopy/int/home.html
VT-iSIM super-resolution microscope (Nikon TiE-based)	VisiTech International	https://visitech.co.uk

### Resource availability

#### Lead contact

Further information and requests for resources and reagents should be directed to and will be fulfilled by the lead contact, Jonathan Higgins (jonathan.higgins@ncl.ac.uk).

#### Materials availability

Cell lines generated in this study are available from the lead contact upon request.

### Experimental model and subject details

#### Cells

HeLa Kyoto cells (female; RRID:CVCL_1922) were grown in Dulbecco’s Modified Eagle’s Medium with 5% (v/v) fetal bovine serum (FBS) and 100 U/ml penicillin-streptomycin. HeLa-TetOn cells expressing Dsn1-EGFP and Dsn1-EE-EGFP (a gift from Soonjoung Kim and Hongtao Yu, University of Texas Southwestern; Kim and Yu, 2015) were grown in similar conditions, with the addition of 150 μg/ml hygromycin B. hTERT-RPE1 cells (female; RRID:CVCL_4388) were grown in DMEM/F12 with 5% (v/v) FBS, and 100 U/ml penicillin-streptomycin. HeLa cells expressing Astrin-EGFP (a gift from Ulrike Gruneberg, University of Oxford; Dunsch et al., 2011) were grown in DMEM with 5% (v/v) FBS and 100 U/ml penicillin-streptomycin with 100 μg/ml G418. To produce a cell line stably expressing Astrin-EGFP and CENP-B-Cherry, HeLa/Astrin-EGFP cells were co-transfected with a 9:1 ratio of pCENP-B-Cherry (a gift from Michael Lampson, University of Pennsylvania; Liu et al., 2010) and pPGKpuro resistance plasmid (Tucker et al., 1996) using X-TremeGENE 9. After selection at 2 μg/ml puromycin, two rounds of cloning by FACS were used to obtain a cell population with consistent moderate levels of Astrin-EGFP and CENP-B-Cherry expression. All cells were maintained in a humidified incubator at 37°C and 5% CO_2_.

### Method details

#### Cell treatments

For anaphase enrichment, in some experiments, cells were treated for 20 h with 2.5 mM thymidine, washed 3 times in pre-warmed PBS, twice in pre-warmed DMEM, and released for 10 h in 5% FBS/DMEM prior to fixation. To induce lagging chromosomes, HeLa and RPE1 cells were treated with 10 μM MPS1-IN-1 for 3 h prior to fixation. For Aurora B inhibition, cells were treated with 1 to 10 μM ZM447439 for 3 to 15 min. To depolymerize anaphase spindles, cells were first blocked in 1 μM nocodazole for 5 h and then, after release for 2 h, treated again with 10 μM nocodazole for 5 min prior to fixation. Dsn1-EGFP expression in HeLa-TetOn cells was induced using 1 μg/ml doxycycline for 20 h.

#### RNA interference

For both Mklp2 and PRC1 depletion, HeLa cells suspended at 0.8 × 10^5^ cells/ml were transfected with 50 nM siRNA using HiPerfect transfection reagent according to the manufacturer’s protocol and analyzed after 48 h.

#### Indirect Immunofluorescence

HeLa cells were grown on poly-L-lysine-coated coverslips. For Dsn1 (19.2B) and Dsn1 S109ph (19.2A/20.2A) staining, cells were pre-extracted in 120 mM PIPES, 50 mM HEPES, 20 mM EGTA, and 8 mM MgSO_4_ pH 7.0 (2x PHEM buffer) containing 1% Triton X-100 for 5 min and then fixed in 4% (w/v) paraformaldehyde in 2x PHEM buffer at 37°C. For Nuf2 staining, cells were fixed in ice-cold methanol for 10 min and then washed in PBS. For Astrin-EGFP staining, cold treatment was carried out by placing cells in L-15 medium with 20 mM HEPES on ice for 10 min, before fixation in 4% w/v paraformaldehyde in 1x PHEM buffer with 0.2% Triton X-100 for 10 min at room temperature. For endogenous Astrin staining, an additional pre-extraction step in 1% Triton X-100 1x PHEM buffer for 5 min at 4°C was carried out. Coverslips were incubated with 1% (w/v) BSA in PBS for 1 h at room temperature, and stained with primary antibody diluted in 1% BSA in PBS for 1 h at 37°C, and secondary antibodies for 45 min at 37°C. Coverslips were mounted on slides using ProLong Diamond with DAPI or ProLong Glass with NucBlue.

#### Fluorescence microscopy

Images of fixed samples were acquired on a Leica SP8 confocal microscope equipped with a 63x 1.4 NA PlanApo Oil objective, capturing data at optimal Nyquist sampling using Leica LasX v3 software, or on a Nikon A1 confocal microscope equipped with a 60x 1.4 NA PlanApo Oil objective, at optimal Nyquist sampling using Nikon Elements 5.22 software, or on a Visitech VT-iSIM super-resolution Nikon TiE-based microscope (Visitech, UK) with instant SIM scanhead coupled to two Hamamatsu Flash4 v2 cameras (Hamamatsu, Japan) via a dual port splitter, using a 100x 1.49 NA PlanApo Oil objective and Nikon Elements 5.21.03 software.

Widefield images were captured with a Zeiss AxioImager microscope equipped with a 63x 1.4 NA PlanApo Oil objective using a Colibri 1 LED light source, an AxioCam MRm camera, and Zeiss ZEN 2.3 software. Optical sectioning was improved by using an Apotome 2 for structured illumination, capturing 3 images per focal plane and channel.

Live imaging was performed in glass-bottomed FluoroDishes (WPI) in Fluorobrite DMEM medium. DNA was stained with 25 nM SiR-DNA (Sen et al., 2018), and ZM447439 was added at double the required final concentration in a volume equal to that of medium in the dish to ensure rapid mixing. Recording was started immediately after drug addition. Images were acquired on a Nikon A1R confocal microscope equipped with a 60x 1.4 NA PlanApo Oil objective, at optimal Nyquist sampling using Elements 5.22 software (Nikon, Japan), or on the Visitech VT-iSIM super-resolution microscope as described above. A humidified environment at 37°C and 5% CO_2_ was maintained inOkolab whole microscope and a stage-top incubators (Okolab, Italy).

### Quantification and statistical analysis

#### Image analysis

Fixed cell image quantification of kinetochore staining was performed using sum intensity projections in Fiji 2.1.0 (Schindelin et al., 2012). The midzone position was defined as the equatorial plane equidistant between the edges of the chromosomes nearest the poles. Circular regions of interest (ROIs) were defined on the ACA or CENP-C channel, and the intensities within each ROI recorded for this and all other channels of interest. The mean of the intensities within 4 background ROIs placed in areas devoid of ACA or CENP-C, but within the chromatin mass, was then subtracted from the relevant channels. Staining intensity at each kinetochore is expressed as a ratio to the ACA or CENP-C intensity at that kinetochore. Dsn1-GFP levels at kinetochores in live cell imaging experiments were quantified using Imaris software, where the DNA channel was used to define the chromatin region within each cell for tracking in time. The intensity of the GFP signal within the defined volume was then recorded for each time point. The mean cytosolic background signal was subtracted from all data points. The rate of chromosome movement in anaphase was quantified using Fiji 2.1.0, where maximum intensity projections of the DNA channel were used to determine the position of the centers of mass of the two groups of chromosomes (x_1_, y_1_ and x_2_, y_2_) at each time point, and the distance from midzone = (√((|x_2_ - x_1_|)^2^ + (|y_2_ - y_1_|)^2^)) / 2. For display purposes only, live images were deconvolved in Nikon Elements using the Richardson-Lucy algorithm. Where noted, fixed cell images were deconvolved with Huygens Software. Normalization, least-squares linear regression, statistical analyses and data visualization were carried out in GraphPad Prism 9.0.1. Statistical details of experiments can be found in the figure legends.

#### Mathematical model

We assumed that there are two populations of Dsn1 associated with kinetochores, one associated with CENP-T and dependent on Cyclin B-Cdk1 (and Aurora B) that is removed rapidly in anaphase, and another associated with CENP-C, and dependent only on Aurora B, that is removed less rapidly in anaphase. The two-phase decay equation was then:

$$Y=B+\left(Y_{0}-B\right)\times F_{Cdk1}\times exp\;\left(-\left(ln\;2/t{½}_{Cdk1}\right)\times t\right)+\left(Y_{0}-B\right)\times F_{AurB}\times exp\;\left(-\left(ln\;2/t{½}_{AurB}\right)\times t\right)$$

The parameters were: *Y* = amount of kinetochore-associated Dsn1; *B* = bottom plateau; *t* = time after anaphase onset; *Y*_*0*_ = Value of *Y* at *t* = 0, *F*_*Cdk1*_ = fraction of Dsn1 loss dependent on Cdk1; *F*_*AurB*_ = fraction of Dsn1 loss dependent on Aurora B; *t½*_*Cdk1*_ = half-life of Cdk1-dependent Dsn1; *t½*_*AurB*_ = half-life of Aurora B-dependent Dsn1. To fit the experimental data, we used least-squares non-linear regression in GraphPad Prism 9.0.1, constraining *Y*_*0*_ = 1, *t½*_*Cdk1*_ to be equal in all conditions, and *t½*_*Cdk1*_ < *t½*_*AurB*_, and assuming that for Dsn1-WT, *F*_*Cdk1*_ = 0.5, *F*_*AurB*_ = 0.5, and for Dsn1-EE that *F*_*Cdk1*_ = 1, *F*_*AurB*_ = 0.

## Data Availability

• All data reported in this paper are available from the lead contact upon request. • Any additional information required to reanalyze the data reported in this paper is available from the lead contact upon request. • This paper does not report original code.

## Supplemental information

Document S1. Figures S1–S5

Document S2. Article plus supplemental information
