# Supplementary material for: The Aurora B gradient sustains kinetochore stability in anaphase
Source: Cell Rep. 2021 Nov 9;37(6):109818. doi: 10.1016/j.celrep.2021.109818 (PMC8595645; doi:10.1016/j.celrep.2021.109818)
Supplement: Document S1. Figures S1–S5 [file mmc1.pdf]

Cell Reports, Volume *37*

Supplemental information

The Aurora B gradient sustains kinetochore stability in anaphase

Diana Papini, Mark D. Levasseur, and Jonathan M.G. Higgins

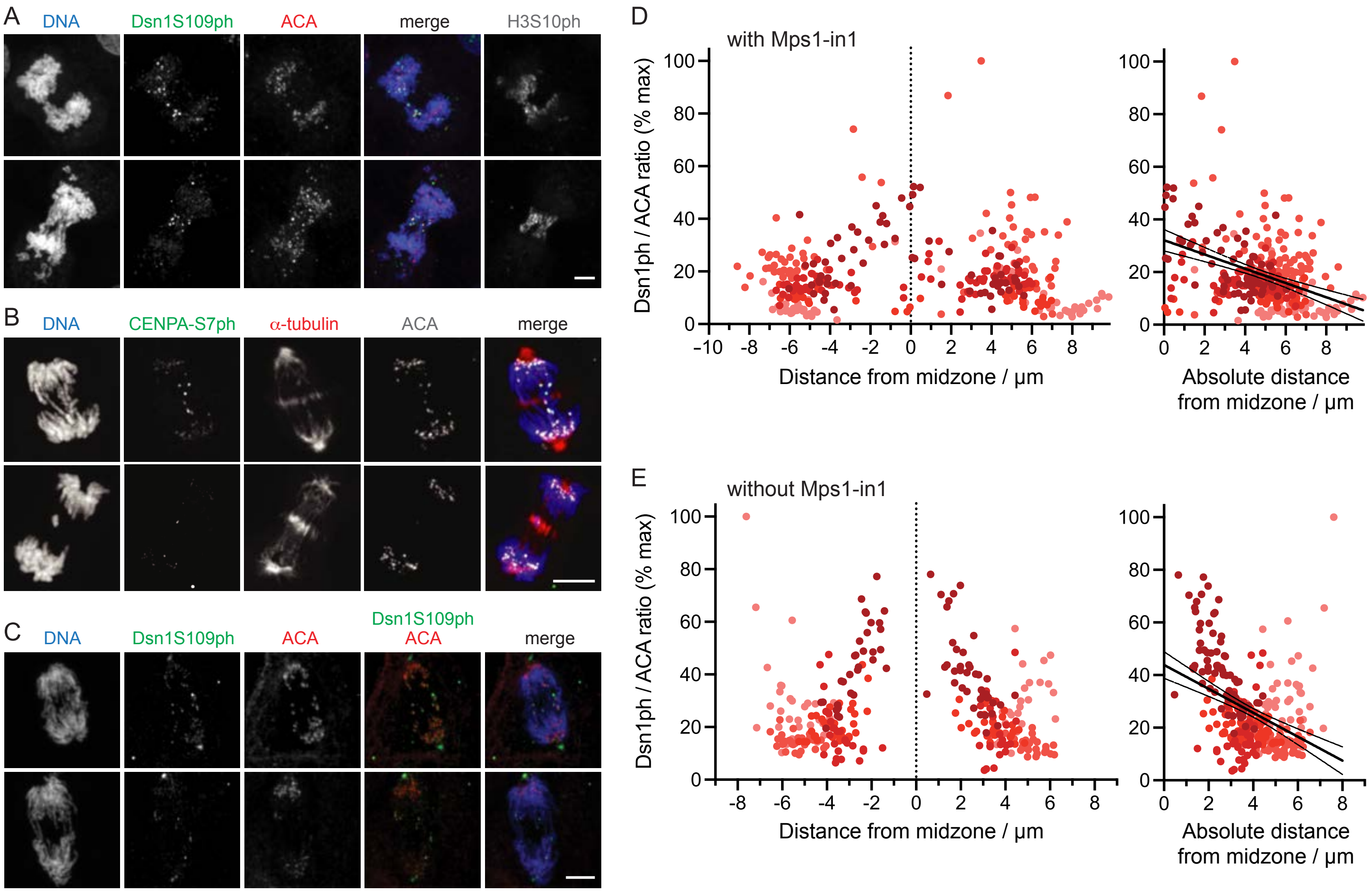

Figure S1. Dsn1S109ph and CENP-AS7ph show anaphase gradients in HeLa and RPE1 cells. Related to Figure 1.
(A) HeLa cells treated with MPS1-IN-1 were stained for DNA (blue), Dsn1S109ph (green, antibody 20.2A), H3S10ph (red), and ACA (gray).
(B) RPE1 cells treated with MPS1-IN-1 were stained for DNA (blue), α-Tubulin (red), CENP-AS7ph (green), and ACA (gray).
(C) RPE1 cells treated with MPS1-IN-1 were stained for DNA (blue), Dsn1S109ph (green), and ACA (red). Channel brightness was adjusted individually for these two cells. Panels A to C, scale bars = 5 μm.
(D) RPE1 cells treated with MPS1-IN-1 were stained as in (C) and Dsn1S109ph was quantified at kinetochores of 5 cells as a function of distance from the midzone. Using linear regression, slope = -2.7 and is non-zero ($p < 0.0001$; F test).
(E) As for (D) but for 5 RPE1 cells not treated with MPS1-IN-1. Using linear regression, slope = -4.5 and is non-zero ($p < 0.0001$; F test). Confidence intervals (95%) shown as fine lines.

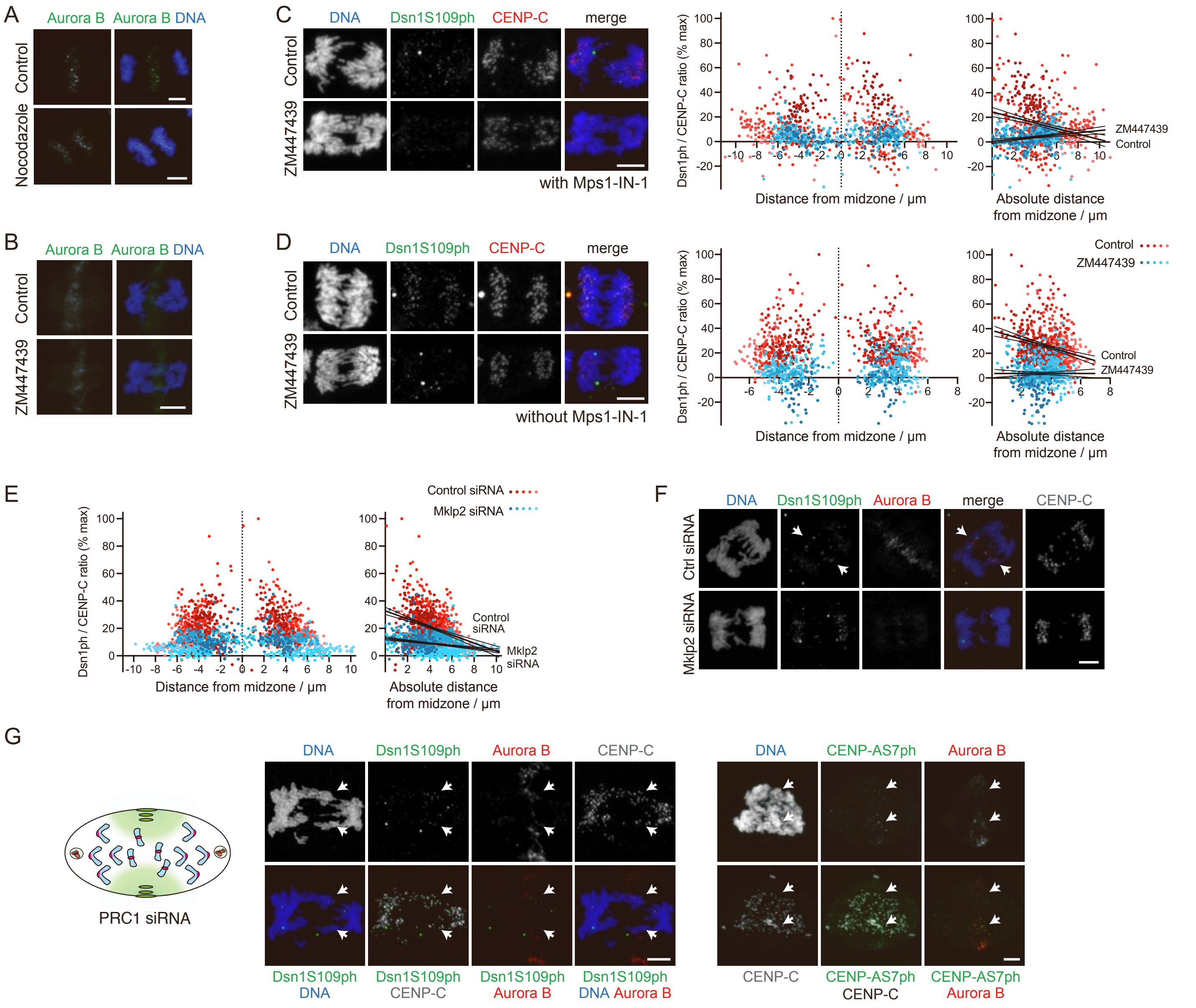

Figure S2. The anaphase Dsn1S109ph gradient is compromised by short term Aurora B inhibition, or depletion of Mklp2 or PRC1. Related to Figure 2.

(A) HeLa cells were treated as in Figure 2A, and stained for DNA (blue) and Aurora B (green) confirming that Aurora B is lost from the midzone upon nocodazole treatment.

(B) HeLa cells were treated as in Figure 2B, and stained for DNA (blue) and Aurora B (green) confirming that (inactive) Aurora B remains at the central spindle upon short term ZM447439 treatment.

(C) HeLa cells were treated with MPS1-IN-1 and then with 1 µM ZM447439 for 3 min prior to fixation and staining for DNA (blue), Dsn1S109ph (green), and CENP-C (red). Dsn1S109ph was quantified at kinetochores in 6 control and 5 ZM447439-treated cells. Using linear regression, for control cells, slope = -2.2 and is non-zero ($p < 0.0001$; F test). For ZM447439-treated cells, slope = 0.9 and non-zero ($p = 0.0001$). The slopes are significantly different from one another ($p < 0.0001$, F test).

(D) As in (C), but for HeLa cells not treated with MPS1-IN-1. Dsn1S109ph was quantified at kinetochores in 7 control and 6 ZM447439-treated cells. Using linear regression, for control cells, slope = -3.5 and is non-zero ($p < 0.0001$; F test). For ZM447439-treated cells, slope = 0.004 and not significantly different from zero ($p = 0.99$). The slopes are significantly different from one another ($p < 0.0001$, F test).

(E) HeLa cells were treated as in Figure 2D, but in the absence of MPS1-IN-1. Dsn1S109ph was quantified at kinetochores in 10 control and 8 Mklp2-depleted cells. Using linear regression, for control cells, slope = -2.9 and is non-zero ($p < 0.0001$; F test). For Mklp2-depleted cells, slope = -0.9 and is non-zero ($p < 0.0001$; F test). The slopes are significantly different from one another ($p < 0.0001$, F test). Confidence intervals (95%) shown as fine lines.

(F) RPE1 cells were transfected with control or Mklp2 siRNA, treated with MPS1-IN-1, and stained for DNA (blue), Dsn1S109ph (green), Aurora B (red) and ACA (gray). Arrows indicate kinetochores with elevated Dsn1S109ph in control cells.

(G) Following PRC1 depletion, the central spindle is disrupted, and Aurora B is retained at the equatorial cortex during anaphase. HeLa cells were transfected with PRC1 siRNA, fixed, and stained for DNA (blue), Dsn1S109ph or CENP-AS7ph (green), Aurora B (red) and CENP-C (gray). Phosphorylation was retained only at those kinetochores in close proximity to Aurora B at the cell cortex (arrows). Note that scattered points of Dsn1S109ph staining that did not overlap with CENP-C staining appear to be due to non-kinetochore background or artifactual staining. Deconvolved images are shown. Panels A to D, F, and G, scale bars = 5 µm.

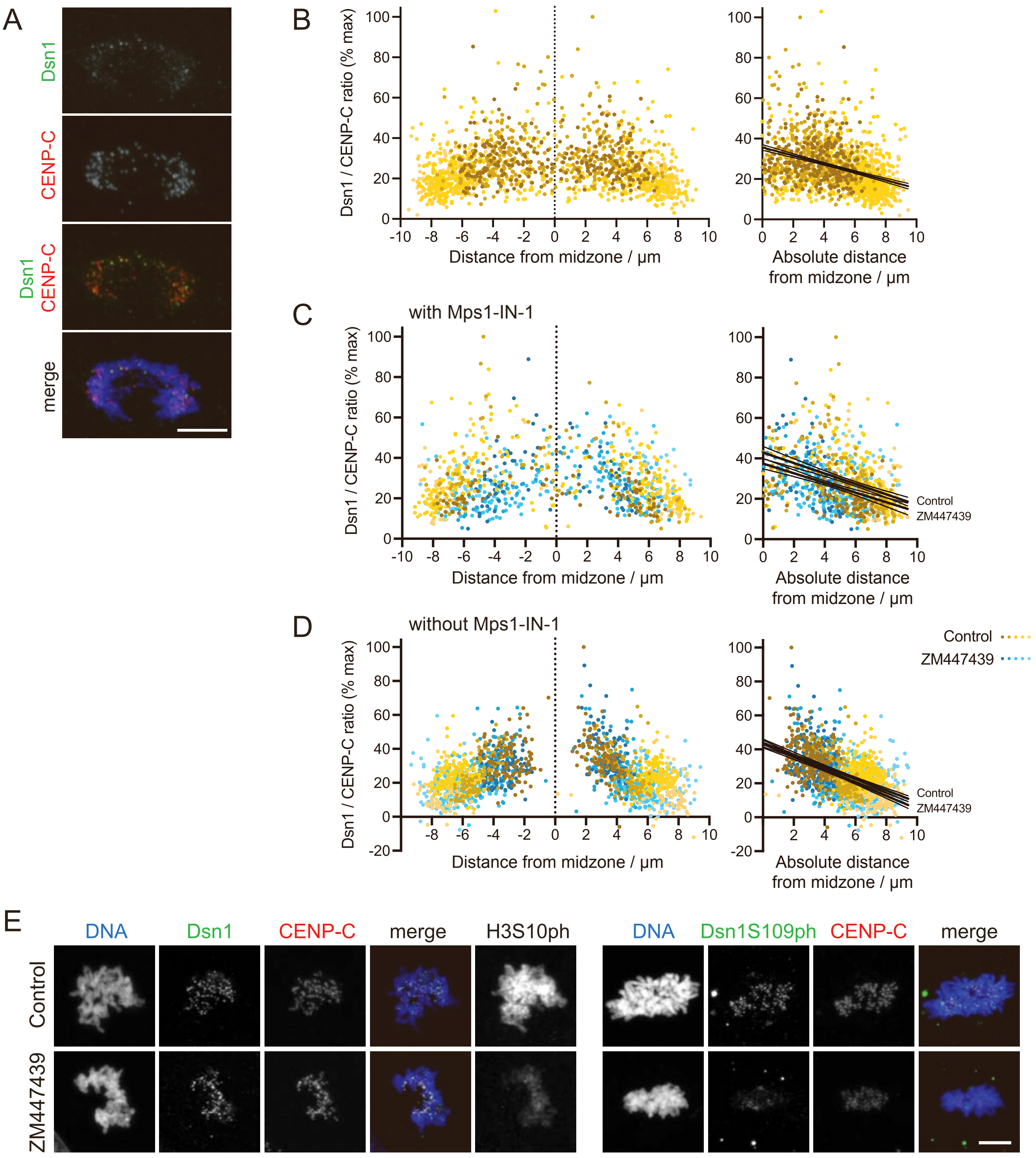

Figure S3. Dsn1 is found in a gradient at anaphase kinetochores. Related to Figure 3.

(A) HeLa cells enriched in anaphases by thymidine-release and treated with MPS1-IN-1 were stained for DNA (blue), total Dsn1 (green), and CENP-C (red).

(B) Quantification of total Dsn1 at kinetochores as a function of distance from the midzone (in 18 cells treated as in A). Using linear regression, slope = -2.0 and is non-zero ($p < 0.0001$; F test).

(C) HeLa cells were treated with Mps1-IN-1 and then with 5 μM ZM447439 for 15 minutes prior to fixation and staining for DNA (blue), Dsn1 (green), and CENP-C (red). Dsn1 was quantified at kinetochores in 6 control and 5 ZM447439-treated cells. Using linear regression for control cells, slope = -2.6, and for ZM447439-treated cells, slope = -2.4, and both are non-zero ($p < 0.0001$; F test). The elevations/intercepts, but not slopes, are significantly different from one another ($p < 0.0001$, F test), suggesting a subtle decrease in Dsn1 localization throughout anaphase in these conditions.

(D) HeLa cells were treated with 5 μM ZM447439 for 5 minutes prior to fixation and staining for DNA (blue), Dsn1 (green), and CENP-C (red). Dsn1 was quantified at kinetochores in 6 control and 5 ZM447439-treated cells. Using linear regression, for control cells slope = -3.5, for ZM447439-treated cells slope = -3.8, and both are non-zero ($p < 0.0001$; F test). Again, the elevations/intercepts, but not slopes, are significantly different from one another ($p < 0.0001$, F test). Confidence intervals (95%) shown as fine lines.

(E) HeLa cells were untreated or treated with 5 μM ZM447439 for 5 minutes prior to fixation and staining for DNA (blue), Dsn1 or Dsn1S109ph (green), CENP-C (red) and H3S10ph (gray). Panels A and E, scale bars = 5 μm.

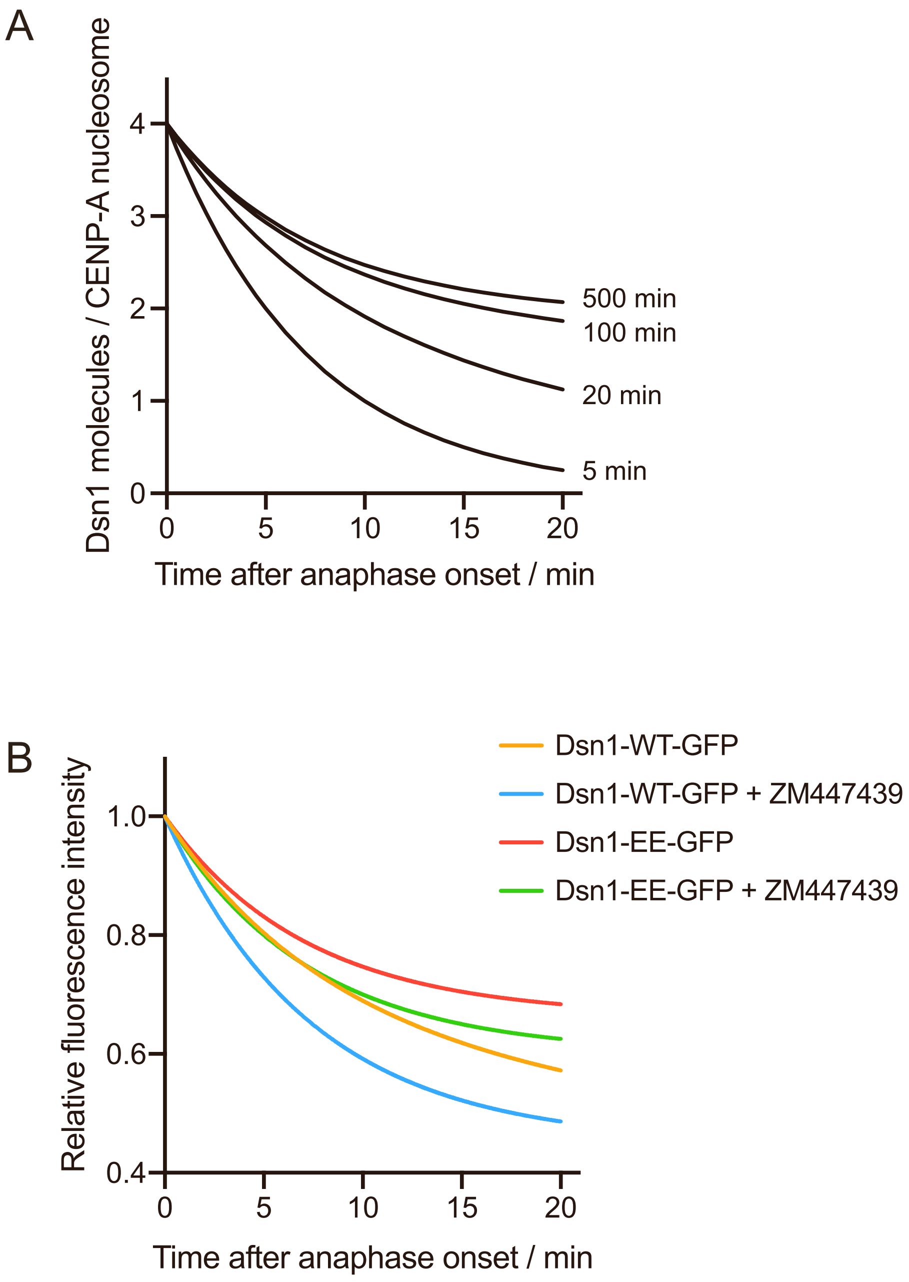

Figure S4. Mathematical model of Dsn1 dissociation from anaphase kinetochores. Related to Figure 4.
(A) The dissociation of Dsn1 from anaphase kinetochores was modelled as a two-phase exponential decay, assuming a starting condition of two molecules of CENP-T-associated Dsn1 and two molecules of CENP-C-associated Dsn1 per centromeric nucleosome(Musacchio and Desai, 2017), and a half-life for Cdk1-dependent Dsn1 dissociation of 5 min. The graph shows the effect of varying the half-life of Aurora B-dependent Dsn1 from 5 to 500 min.
(B) Least squares linear regression fit of the two-phase exponential decay model to the data in Figure 4C (see Methods).

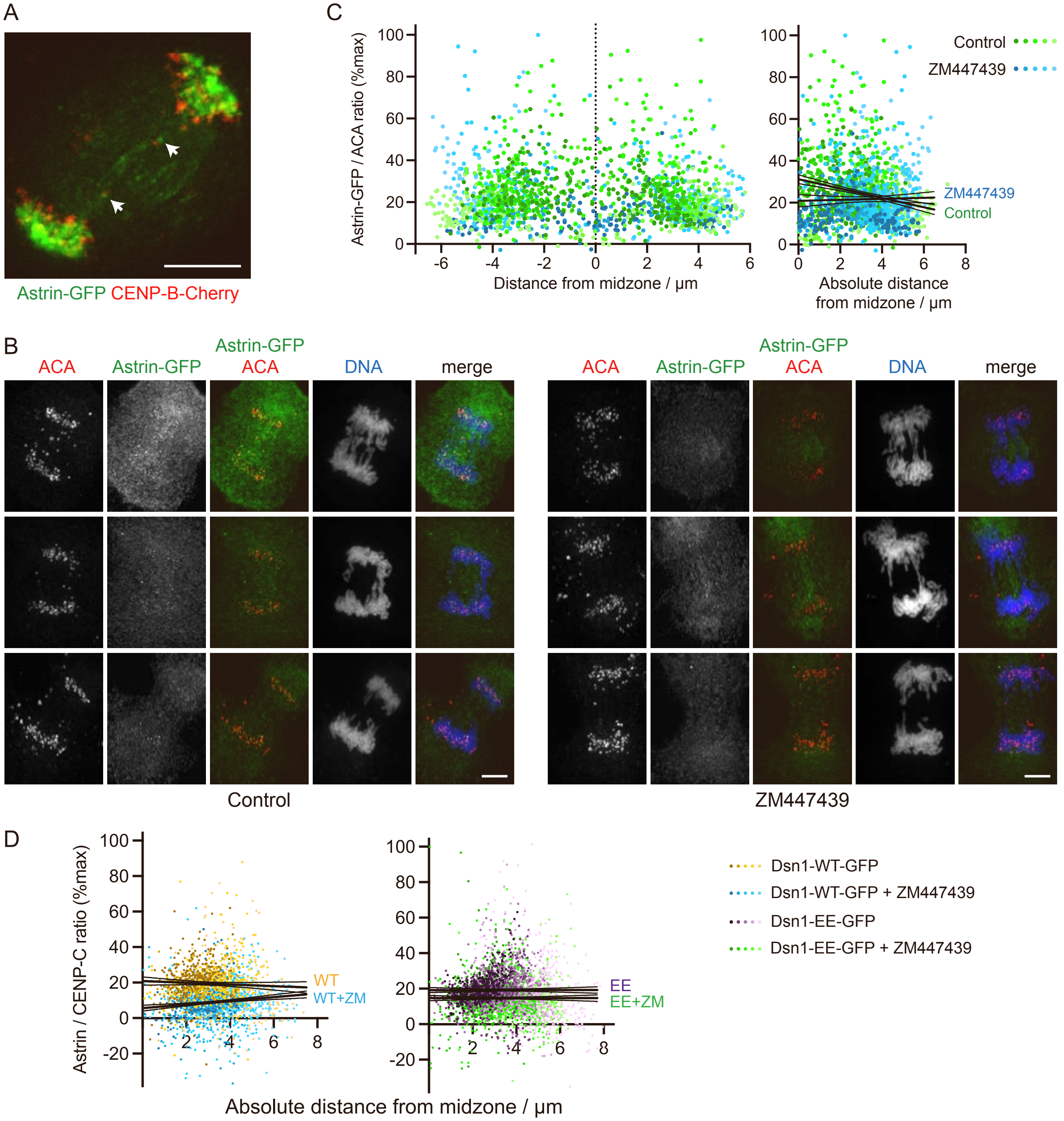

Figure S5. Astrin localization in anaphase is influenced by Aurora B. Related to Figure 6.
(A) A living HeLa cell stably expressing Astrin-GFP (green) and CENP-B-Cherry (red) was imaged by iSIM super-resolution microscopy. Lagging kinetochores that maintain Astrin-GFP are indicated with white arrows. See also Video S7.
(B) MPS1-IN-1-treated HeLa cells expressing Astrin-GFP were exposed to 5 µM ZM447439 or control for 10 min, then cold treated to depolymerise labile microtubules, fixed and stained for DNA (blue), GFP (green), and ACA (red). Deconvolved images are shown. Panels A and B, scale bars = 5 µm.
(C) For cells treated as in (A), Astrin-GFP at kinetochores was quantified as a function of distance from the midzone. Using linear regression, for 19 control cells, slope = -2.2 which is non-zero ($p<0.0001$, F test), and for 11 ZM447439-treated cells, slope = 0.24 which is not significantly different from zero ($p = 0.57$; F test). The slopes are significantly different from one another ($p < 0.0001$, F test). Confidence intervals (95%) shown as fine lines.
(D) Aurora B inhibition weakens Astrin localization to early anaphase kinetochores in cells expressing Dsn1-WT-GFP, but less so in cells expressing Dsn1-EE-GFP. In anaphase HeLa cells expressing Dsn1-WT-GFP or Dsn1-EE-GFP, endogenous Astrin at individual kinetochores was quantified as a function of distance from the midzone. Using linear regression, for 15 Dsn1-WT-GFP cells, 22 ZM447439-treated Dsn1-WT-GFP cells, 22 Dsn1-EE-GFP cells, and 22 ZM447439-treated Dsn1-EE-GFP cells, the slopes are -0.5, 1.0, 0.006, and -0.005, respectively. Comparing Astrin in Dsn1-WT-GFP cells treated with and without 10 µM ZM447439 for 15 min, the slopes are significantly different from one another ($p = 0.0002$, F test). Confidence intervals (95%) shown as fine lines.
